# Assessing the relationship between women's attitudes toward food waste prevention and their green diets

**DOI:** 10.3389/fnut.2025.1630098

**Published:** 2025-12-01

**Authors:** Sevinç Eşer Durmaz, Aylin Bayındır Gümüş, Fatma Nişancı Kılınç, Cansu Ergenç Özdaş

**Affiliations:** 1 Department of Nutrition and Dietetics, Faculty of Health Sciences, Kirikkale University, Kirikkale, Türkiye; 2 First and Emergency Aid Program, Vocational School of Health Services, Kirikkale University, Kirikkale, Türkiye; 3 Faculty of Business Administration, Banking and Finance, Statistics, Ankara Yildirim Beyazit University, Ankara, Türkiye

**Keywords:** eco-friendly, environment, food waste, green eating, obesity, sustainability

## Abstract

**Background:**

Food waste is one of the major obstacles to sustainable nutrition. The aim of this study is to examine the relationship between women's attitudes and behaviors toward preventing food waste and their green eating behaviors.

**Methods:**

This study was conducted with 870 adult women aged 19–64 across Türkiye. Data collection was conducted in person through face-to-face interviews. A comprehensive questionnaire was administered to participants during the interviews. The questionnaire included questions on demographic and socioeconomic characteristics, green eating (GE), attitudes and behaviors toward preventing food waste (FWP), frequency of food waste from various food groups, frequency of food waste prevention actions (FWP-FA), and several anthropometric measurements [such as height, weight, waist circumference, and body mass index (BMI)]. Data obtained in the study were analyzed using the SPSS 29.0 statistical package.

**Results:**

The FWP mean scores of the participants are low in the 19 and 29 age group. The mean scores of GE [*F*_(4, 865)_ = 2.338, *p* = 0.05] and FWP [*F*_(4, 865)_ = 3.272, *p* = 0.011] differ significantly according to educational level. According to income levels, both GE mean scores [*F*_(2, 865)_ = 4.298, *p* = 0.01] and FWP mean scores [*F*_(2, 865)_ = 3.154, *p* = 0.043] differ significantly. While GE [*F*_(3, 863)_ = 0.836, *p* = 0.47] mean scores did not differ according to BMI levels, FWP [*F*_(3, 863)_ = 5.731, *p* = 0.001] mean scores differed significantly according to BMI levels.

**Conclusions:**

The findings indicate that FWP has a significant positive effect GE, and that the combined influence of FWP and FWP-FA on GE is both strong and statistically significant. Additionally, waist circumference was found to have a small but significant negative impact on GE. Although BMI was analyzed as a potential mediator, it did not significantly mediate the relationship between FWP and GE. These results suggest that attitudes and behaviors aimed at reducing food waste are directly associated with green eating practices, while obesity-related indicators such as waist circumference may negatively influence this relationship.

## 1 Introduction

Food waste is considered one of the most serious sustainability challenges at the global level today. The continuous increase in the human population brings a growing demand for food and food products, which in turn leads to an increase in agricultural activities and food waste (1). Waste occurs at all stages of the food supply chain, yet it is particularly concentrated at the consumer level, a phenomenon directly related to individuals' dietary preferences (2). Food waste is not only an economic issue but also has significant environmental consequences. It is recognized as a critical threat to food security, the economy, and the environment (3). Each wasted food item represents the loss of valuable resources used during production such as water, energy, land, and labor, while also contributing to increased greenhouse gas emissions and climate change (4, 5). Among the impacts of food waste are the inefficient use of water and land resources, unnecessary greenhouse gas emissions, and the degradation of natural ecosystems and the services they provide (4). Combating food waste is a crucial step toward achieving sustainability. According to the food waste hierarchy, preventing food waste is the top priority due to its significant environmental benefits, as demonstrated by life cycle assessments (6). Nutrition plays an important role in preventing food waste. Conscious eating habits prevent waste by ensuring proper planning, storage and evaluation of food (5).

Most research on consumer behavior has focused on explaining the high levels of food waste at the household level and proposing solutions to reduce food waste by consumers (3, 7). It is observed that women play an important role in preventing food waste, and this may be related to their taking on more responsibility in the kitchen due to their gender roles (8). On the other hand, studies on environmental concerns have significantly advanced in recent years to understand the role of these factors in explaining environmental behavior (9).

Considering the active role of consumers—especially women—in food management within the household, investigating the relationship between food waste prevention behaviors and green eating tendencies has become an important area of research (8). Women play a critical role in household food planning, purchasing decisions, and waste management processes, which places them at the center of adopting sustainable dietary behaviors and reducing food waste.

Green eating is emerging as a key component of a sustainable nutrition approach. This concept offers a holistic approach that encompasses not only low environmental impact but also nutritious food choices, fair trade practices, biodiversity conservation, and support for local food systems. In this context, the concept of green eating (GE) is defined as a socially and environmentally conscious dietary pattern. It includes primarily plant-based eating, limiting red meat consumption, choosing local and seasonal foods, consuming organic products, and avoiding highly processed foods (10, 11). Studies have shown that plant-based diets are more efficient in terms of water and energy use and have a lower environmental footprint compared to meat-based diets (12, 13).

Improving nutritional quality while reducing environmental impact and achieving sustainable development goals has become a critical global focus (14). Consumers play a key role in transitioning the food system toward sustainability by adopting environmentally and socially responsible eating behaviors, also referred to as green eating (11). Green eating includes behaviors such as limiting red meat consumption, following a primarily plant-based diet, reducing intake of high-fructose corn syrup, eating organic foods, and shopping locally—all of which are associated with lower environmental impacts (10). Research indicates that primarily plant-based diets are more environmentally sustainable than meat-based diets due to lower fossil fuel and water usage (12, 13). Such dietary patterns are part of a broader shift toward more eco-friendly and sustainable eating behaviors (11).

The aim of this study is to examine the relationship between women's attitudes and behaviors toward preventing food waste and their green eating behaviors, and to evaluate the potential mediating effect of body mass index (BMI) in this relationship. The findings are expected to contribute to the development of strategies for sustainable nutrition.

The conceptual framework of this research examines the effects of women's attitudes and behaviors toward preventing food waste on green eating. First, it is hypothesized that these attitudes and behaviors directly affect green eating (H1). Furthermore, it is anticipated that this relationship may occur not only directly but also indirectly. In this context, variables such as frequency of food waste prevention actions, waist circumference, and body mass index (BMI) may act as mediators, explaining the impact of individuals' environmental sensitivity on their dietary behaviors (H2, H3, H4).

Furthermore, it is anticipated that these mediation effects may differ depending on individual demographic factors. It is hypothesized that age and income level will conditionally shape the indirect effects on green eating through frequency of food waste prevention actions, waist circumference, and BMI (H5–H10). Thus, the conceptual model suggests that environmental sensitivity may play a determining role not only in individuals' attitudes and behaviors but also in green eating habits through health indicators and demographic factors.

### 1.1 Direct effect hypothesis

H1: There is a significant direct effect of the Attitude and Behavior Toward Prevention of Food Waste on Green Eating.

### 1.2 Mediation hypotheses

H2: The Frequency of Actions to Prevent Food Waste mediates the relationship between the Attitude and Behavior Toward Prevention of Food Waste and Green Eating.
H3: Waist circumference mediates the relationship between the Attitude and Behavior Toward Prevention of Food Waste and Green Eating.
H4: Body Mass Index mediates the relationship between the Attitude and Behavior Toward Prevention of Food Waste and Green Eating.

### 1.3 Moderated mediation hypotheses

H5: The indirect effect of the Attitude and Behavior Toward Prevention of Food Waste on Green Eating through the Frequency of Actions to Prevent Food Waste is conditional on age.
H6: The indirect effect of the Attitude and Behavior Toward Prevention of Food Waste on Green Eating through the Frequency of Actions to Prevent Food Waste is conditional on income.
H7: The indirect effect of the Attitude and Behavior Toward Prevention of Food Waste on Green Eating through waist circumference is conditional on age.
H8: The indirect effect of the Attitude and Behavior Toward Prevention of Food Waste on Green Eating through waist circumference is conditional on income.
H9: The indirect effect of the Attitude and Behavior Toward Prevention of Food Waste on Green Eating through BMI is conditional on age.
H10: The indirect effect of the Attitude and Behavior Toward Prevention of Food Waste on Green Eating through BMI is conditional on income.

This study aims to explain sustainable food consumption behaviors in a holistic way by examining the direct effect of attitudes and behaviors toward preventing food waste on green eating in women (H1), the mediating roles of the frequency of food waste prevention actions (H2), waist circumference (H3) and body mass index (H4) in this relationship, and how these indirect effects vary depending on socio-demographic factors such as age (H5, H7, H9) and income level (H6, H8, H10).

## 2 Materials and methods

### 2.1 Participants

Participants were adult, healthy women who voluntarily agreed to participate in the study. Informed consent was obtained from the individuals who participated in the study. No financial incentives were given to the participants. The study lasted 10 min for each individual. There were no open-ended questions in our study. The widely accepted G-power analysis was used to determine the sample size (15). We conducted a study to select a sufficient sample size to detect a moderate effect size (Cohen's *d* = 0–0.2) (16, 17). A sensitivity power analysis with a power of 1 – β = 0.95, a Type I error rate of α = 0.05, and a Cohen's *d* effect size of 0.15 indicated a sample size of 567. Increasing the sample size reduces the amount of sampling error and results in smaller confidence intervals; this increases statistical power (18–20). To ensure scientific validity and address ethical considerations, a sufficient sample size was determined, and a total of 870 participants were selected based on factors such as cost, accessibility, and the quality of both correct and incorrect survey responses.

The survey consisted of sociodemographic characteristics, green eating behavior scale, a scale of attitudes and behaviors toward preventing food waste, and some anthropometric measurements.

In this study, the following data were collected: age, education level, economic level, total number of people living in the household, occupation, Existence NCDs BMI classification, and waist circumference.

Data were collected between September and December 2023. The study was conducted in accordance with the principles of the Declaration of Helsinki. Necessary permissions were obtained from the original authors of the adapted measurement scales used in the study. The study was approved by the Non-Interventional Research Ethics Committee of Kirikkale University at its meeting dated September 27, 2023 (Protocol No. 422269). Participants were informed about the purpose and content of the study, and written and verbal informed consent was obtained from them.

### 2.2 Anthropometric measurements

Body mass index (BMI) values of the individuals were calculated using the formula [BMI = BW (kg)/height^2^ (m^2^)], which is obtained by dividing body weight in kilograms by the square of height in meters. According to the classification defined by the World Health Organization (WHO), individuals with a BMI below 18.5 kg/m^2^ are classified as underweight, those between 18.5 and 24.9 kg/m^2^ as normal weight, between 25.0 and 29.9 kg/m^2^ as overweight, and those with a BMI of 30.0 kg/m^2^ or above as obese (21). Waist circumference measurements were conducted while participants were in a standing position, using a non-elastic (rigid) measuring tape in direct contact with the skin, following standardized measurement protocols. Measurements were recorded in centimeters (cm). According to WHO classification, individuals with a waist circumference of ≥80 cm and < 88 cm are considered to be at increased metabolic risk, while those with a waist circumference of ≥88 cm are classified as being at high risk (21). These criteria are widely used as reference indicators in the assessment of cardiometabolic disease risks associated with abdominal obesity (21).

### 2.3 Green eating behavior scale (GE)

The scale is a tool developed by Weller et al. (10) that assesses environmentally responsible eating behavior (10). The validity and reliability of Turkish version, which was carried out by Cambaz (22). The scale consisted of five items in which respondents could indicate how often they consumed green food products on a five-point scale ranging from “Never” (=1) to “Always” (=5) or “I don't know” The scale consists of four subscales (Green eating stage of change, Green eating behavior, decisional balance, self-efficacy) and 25 items. Overall Cronbach alpha was found as 0.77. The scale is a five-point Likert type and the total score is calculated by adding the scores and dividing by the number of items.

### 2.4 Attitude and behavior for preventing food waste (FWP)

This tool was developed by Likert (23). It is based on the principle that the participants determine their positive, negative or neutral agreement with each of the statements in the item set created to measure a certain construct. In the literature, Likert-type scales have been used to measure attitudes and behaviors in many studies on food waste of consumers. The validity and reliability of the scale items created by Daysal (24) were carried out. The scale is evaluated as a 5-point Likert scale (1: never, 2: rarely, 3: sometimes, 4: often, 5: always) (24).

### 2.5 Frequency of wasting food groups

In order to examine the frequency of women's wasting of various foods were divided into 7 groups: milk group, meat group, bread and cereals group, fresh vegetables and fruits group, cooked meals, oils and packaged/junk foods. The frequency of consumers' wasting of these products was evaluated as not consuming (0), never (1), rarely (2), sometimes (3), often (4), always (5) (25).

### 2.6 Frequency of actions to prevent food waste (FWP-FA)

Actions to prevent food waste were created by the researchers as a result of the literature review. Responses were scored as 1 no, 2 sometimes, 3 yes (4, 26).

### 2.7 Statistical analysis

SPSS (Statistical Package for the Social Sciences) package program (IBM SPSS Statistics 29.0. Armonk, NY, USA: IBM Corp; 2013) was used to estimate descriptive, correlation statistics and the Hayes model. Before the statistical analysis, assumptions (normality, multicollinearity, and autocorrelation) were verified. The distribution of the variables was investigated, and it was seen that the skewness and kurtosis values of each variable were less than ±1.5 (27). Independent *T*-test and One-Way ANOVA were applied to examine whether GE and FWP differed in terms of socio-demographic values. Differences between groups were examined with Tukey and LSD *post-hoc* tests. The mean difference is significant at the ≤ 0.05 level. Confirmatory factor analysis based on was used to assess the validity and reliability of Green Eating Survey in women. In this study, confirmatory factor analysis (CFA) was applied to comprehensively evaluate the validity and reliability of the Green Eating (GE) Scale specific to the female population. In the analysis, the pre-defined factor structure was tested and the following psychometric properties were examined. Construct validity was assessed: Standardized factor loadings (λ > 0.5, *p* < 0.01), Model fit: Goodness of fit indices (χ^2^/sd < 3, CFI > 0.90, RMSEA < 0.08), Internal consistency: Cronbach's alpha (>0.70) and composite reliability (CR > 0.70), Convergent validity: Average explained variance (AVE > 0.50). CFA was conducted using the maximum likelihood method in the SPSS AMOS program, ensuring the suitability of the scale for measuring women's green nutrition behaviors in the Turkish cultural context. Hayes model 9 was applied using 5,000 bootstrap samples with 95% confidence intervals to validate the proposed hypotheses (28).

## 3 Results

According to the sociodemographic characteristics of the women, food waste prevention and Green Eating is shown in Table 1. The ANOVA results showed that mean GE scores did not differ according to age groups [*F*_(4, 863)_ = 1.117, *p* = 0.34], yet mean FWP scores significantly differed [*F*_(4, 863)_ = 9.633, *p* < 0.001]. It is seen that the behaviors of the participants toward preventing food waste were low in the 19–29 age range. According to the results of ANOVA analysis, the mean scores of GE [*F*_(4, 865)_ = 2.338, *p* = 0.05] and FWP [*F*_(4, 865)_ = 3.272, *p* = 0.011] differ significantly according to educational level. Education level was an effective factor in preventing food waste and increasing Green Eating behavior. According to income levels, both GE mean scores [*F*_(2, 865)_ = 4.298, *p* = 0.01] and FWP mean scores [*F*_(2, 865)_ = 3.154, *p* = 0.043] differ significantly. It was seen that the mean scores of the behaviors toward preventing food waste of individuals with low income level were higher. Individuals with higher income level had higher Green Eating behavior scores. The mean scores of GE [*F*_(5, 864)_ = 0.941, *p* = 0.489] and FWP [*F*_(5, 864)_ = 1.329, *p* = 0.217] did not differ according to the number of people living in the household. While GE mean scores did not differ on occupation type [*F*_(5, 864)_ = 1.943, *p* = 0.08], it showed a marginal difference on FWP mean scores [*F*_(5, 864)_ = 2.143, *p* = 0.05]. It is seen that the behaviors of officer to prevent food waste are higher compared to other professions. While GE [F_(3, 863)_ = 0.836, *p* = 0.47] mean scores did not differ according to BMI levels, FWP [*F*_(3, 863)_ = 5.731, *p* = 0.001] mean scores differed significantly according to BMI levels. It was determined that overweight and obese individuals had high behavior scores for preventing food waste. Waist circumference groups do not have significant differences on GE [*F*_(2, 853)_ = 0.811, p = 0.445] and FWP [*F*_(2, 853)_ = 2.421, p = 0.089].

**Table 1:** GE and FWP Scores according to sociodemographic characteristics and BMI groups of the women.

**Features**	***n* (%)**	**GE**	**FWP**	**GE**	**FWP**	**GE**	**FWP**
	***n** =* **870**	***F***	***F***	***t***	**t**	***p***	***p***
**Age groups (years)**		1.117	9.633			0.34	< 0.001
19–29	395 (45.4%)						
30–39	255 (29.3%)						
40–49	158(18.2%)						
50–59	54 (6.2%)						
60–64	8 (0.9%)						
**Education status**		2.338	3.272			0.05	0.011
Primary school	28 (3.2%)						
Middle school	43 (4.9%)						
High school	190 (21.8%)						
Bachelor	554 (63.7%)						
Master	55 (6.3%)						
**Income level**		4.298	3.154			0.01	0.04
Lower income	207 (23.8%)						
Moderate income	445 (51.1%)						
High income	218 (25.1%)						
**Total people living in the household**		0.941	1.329			0.48	0.21
1	66 (7.6%)						
2	130 (14.9%)						
3	217 (24.9%)						
4	281 (32.4%)						
5	127 (14.6%)						
6>	49 (5.6%)						
**Occupation**		1.943	2.143			0.08	0.05
Officer	341 (39.2%)						
Employee	100 (11.5%)						
Small business	27 (3.1%)						
Private sector	278 (32.0%)						
Self-employment	56 (6.4%)						
Other^*^	68 (7.8%)						
**Existence NCDs**				0.506	2.629	0.61	0.009
Yes	110 (12.6%)						
No	760 (87.4%)						
**BMI classification**		1.254	8.330			0.47	0.001
Underweight	61 (7.01%)						
Normal weight	481 (55.29%)						
Overweight	233 (26.78%)						
Obese	95 (10.92%)	0.811	2.421			0.445	0.089
**Waist circumference**							
Normal (< 80)	547 (62.8%)						
Risk (80–87 cm)	143 (16.4%)						
High risk (≥88 cm)	180 (20.6%)						

GE, Green eating; FWP, Attitude and Behavior Scale for Preventing Food Waste; NCDs, non-communicable disease; BMI, body mass index.

**Figure 1:**
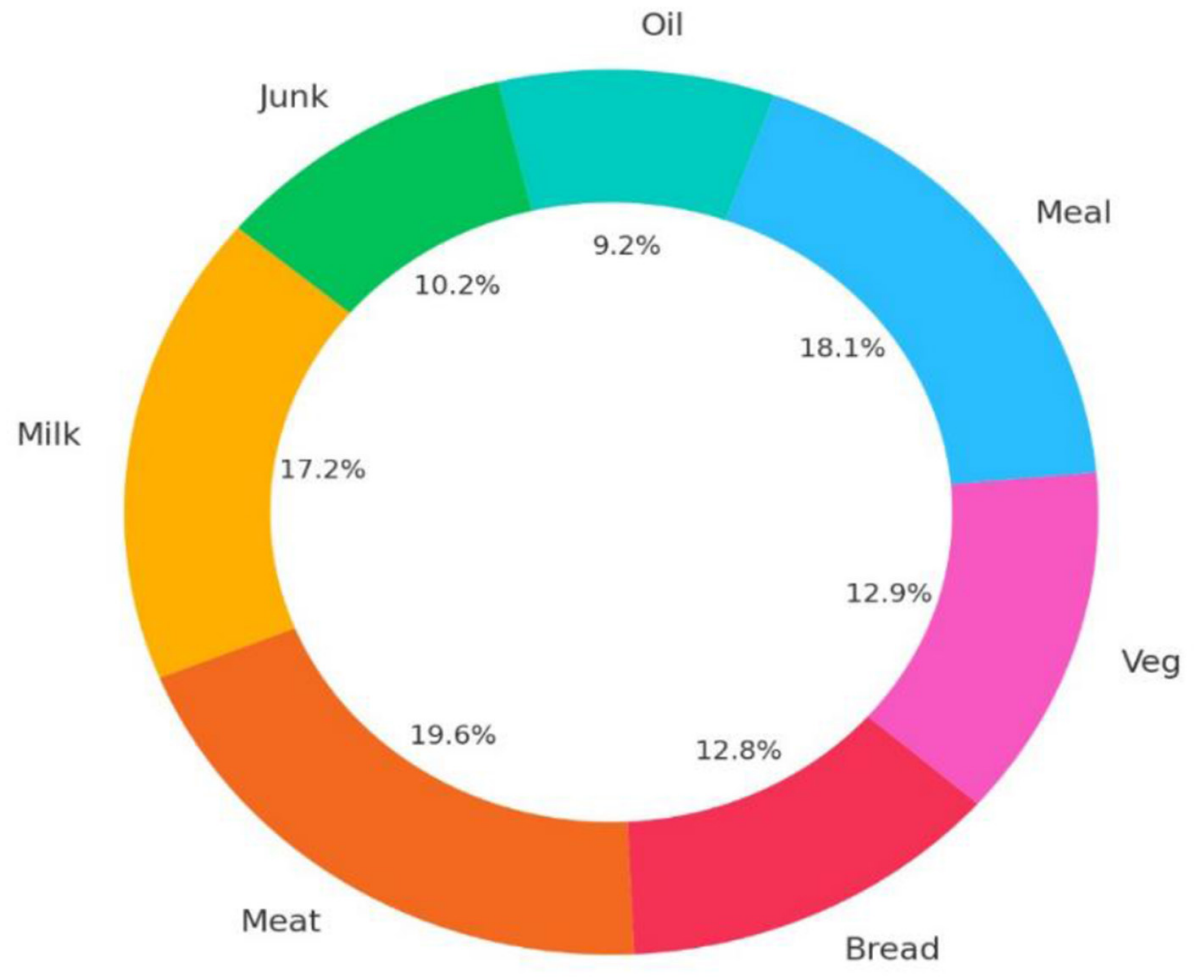
Frequency of women wasting food groups.

In the study, the frequency of women wasting various food groups was evaluated in Figure 1. It was determined that they wasted meat and eggs (%19,6), cooked meals (%18,1), and milk and dairy products (%17,2), respectively.

Table 2 shows Factor loadings, Cronbach's a, CR and AVE scores for GE in women. Factors 1, 2, and 3 represent Green Eating Behavior (Items 1, 2, 3, 4, 5, 6), Decision Making for Green Eating (Items 7, 8, 9, 10, 11, 12, 13, 14, 15, 16), and Self-Efficacy for Green Eating (Items 17, 18, 19, 20, 21, 22, 23), respectively. The CR value was 0.949, which significantly exceeds the 0.70 threshold. This indicates that the reliability of the structures was high. CR was a measure of the overall reliability of a collection of heterogeneous but similar items and provided a more accurate measure of reliability than Cronbach's α. The AVE of the primary variable set was 0.440. Although AVE values greater than 0.5 were preferred as they indicate that more than half of the variance in the indicators is explained by the latent construct, values slightly below this threshold may still be acceptable depending on the context.

**Table 2:** Factor loadings, Cronbach's *a*, CR and AVE scores of GE.

**Variables**	**Factor loadings**	**Cronbach's α**	**CR**	**AVE**
Factor 1: Green eating behavior	0.568–0.767	0.874		
Factor 2: Decision making for green eating	0.510–0.724		0.949	0.440
Factor 3: Self-efficacy for green eating	0.668–0.785			

CR, composite reliability; AVE, average variance extracted; Cronbach's α > 0.70; CR > 0.70; CR > AVE.

The R-squared values show that the models account for between 14.09% and 25.72% of the variability in the outcome variables. Also, the F-statistics and *p*-values (< 0.001 for all models) shows that all models were statistically significant (Table 3).

**Table 3:** Model summary for each outcome variable.

**Outcome Variable**	***R***	***R* ^2^**	**MSE**	***F***	**d*f*1**	**d*f*2**	***p***
FWP	0.5072	0.2572	0.0675	58.1857	5	840	0.0000
GE	0.3754	0.1409	0.3378	34.4917	4	841	0.0000
Waist circumference	0.3934	0.1547	144.1561	30.7526	5	840	0.0000
BMI	0.3976	0.1581	19.7854	31.5482	5	840	0.0000

**Figure 2:**
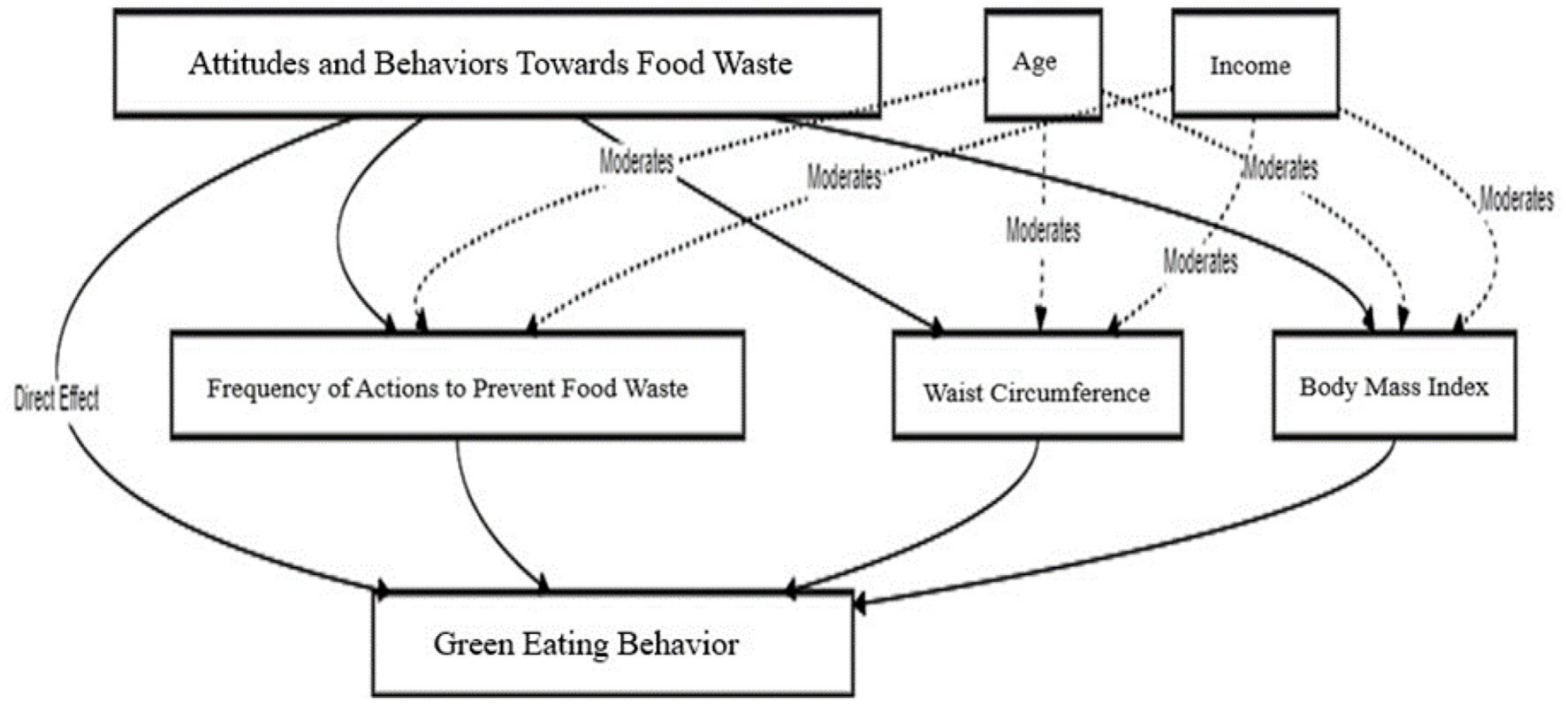
Relationship of food waste prevention and Green Eating behavior with a mediation analysis.

The coefficients for each outcome variable are presented in Table 4 and relationship of food waste prevention and green eating behavior with a mediation analysis shown in Figure 2. Attitude and Behavior Scale Toward Prevention of Food Waste on GE is positively significant (*B* = 0.2117, *p* < 0.00). Therefore, H1 hypothesis was accepted. The Frequency of Actions to Prevent Food Waste has a strong, positive and significant effect on GE (*B* = 0.5409, *p* < 0.001). There was a positive and significant effect between FWP and FWP-FA (*B* = 0.4154, *p* < 0.001), indicating that as FWP increased, FWP-FA also increased. Waist circumference has a small negative yet significant effect on GE (*B* =−0.00041, *p* = 0.0418), indicating that GE decreased as waist circumference increases. The relationship between BMI and GE appears to be negative but not statistically significant. The indirect effect of FWP on GE via FWP-FA was significant across all ages and income levels, as indicated by the positive effects and the confidence intervals that do not include zero. Therefore, H2 hypothesis was accepted. The indirect effect of FWP on GE via waist circumference was not significant, as indicated by the confidence intervals that include zero across all age and income levels. This suggests that waist circumference did not mediate the relationship between FWP and GE. H3 hypothesis was not accepted.

**Table 4:** Coefficients for each outcome variable.

**Predictor**	**Coeff**	**SE**	***t***	***p***	**LLCI**	**ULCI**
**Outcome: FWP-FA**						
Constant	0.6183	0.2992	2.0664	0.0391	0.0310	1.2055
FWP	0.4154	0.0770	5.3969	0.0000	0.2643	0.5664
Age	0.0107	0.0083	1.2909	0.1971	−0.0056	0.0269
FWP × Age	−0.0020	0.0021	−0.9781	0.3283	−0.0061	0.0020
Income	0.0000	0.0000	1.4574	0.1454	0.0000	0.0000
FWP × Income	0.0000	0.0000	−1.4855	0.1378	0.0000	0.0000
**Outcome: GE**						
Constant	1.4940	0.2154	6.9364	0.0000	1.0713	1.9168
FWP	0.2117	0.0483	4.3830	0.0000	0.1169	0.3065
FWP-FA	0.5409	0.0767	7.0519	0.0000	0.3904	0.6915
Waist circumference	−0.0041	0.0020	−2.0382	0.0418	−0.0080	−0.0002
BMI	−0.0008	0.0054	−0.1428	0.8865	−0.0115	0.0099
**Outcome: waist circumference**						
Constant	62.6597	13.8238	4.5328	0.0000	35.5265	89.7929
FWP	−0.3012	3.5561	−0.0847	0.9325	−7.2810	6.6786
Age	0.4439	0.3821	1.1618	0.2457	−0.3061	1.1940
FWP × Age	0.0225	0.0955	0.2356	0.8138	−0.1650	0.2100
Income	0.0000	0.0002	0.1428	0.8865	−0.0003	0.0003
FWP × Income	0.0000	0.0000	−0.3217	0.7478	−0.0001	0.0001
**Outcome: BMI**						
Constant	17.1643	5.1213	3.3515	0.0008	7.1122	27.2164
FWP	0.4688	1.3174	0.3558	0.7221	−2.1170	3.0546
Age	0.1017	0.1416	0.7186	0.4726	−0.1761	0.3796
FWP × Age	0.0219	0.0354	0.6179	0.5368	−0.0476	0.0913
Income	0.0001	0.0001	1.0510	0.2936	−0.0001	0.0002
FWP × Income	0.0000	0.0000	−1.3847	0.1665	−0.0001	0.0000

n = 870; GE, Green eating; BMI, Body mass index; FWP, Attitude and behavior scale for preventing food waste; FWP-FA, Frequency of actions to prevent food waste; LLCI, Lower limit of the confidence interval; ULCI, Upper limit of the confidence interval.

The indirect effect of FWP on GE via BMI was not significant, as indicated by the confidence intervals that include zero across all age and income levels. This suggests that BMI did not mediate the relationship between FWP and GE. H4 hypothesis was not accepted. The index of moderated mediation for FWP-FA with respect to age was not significant, as the confidence interval includes zero. This suggests that age did not significantly moderate the mediation effect of FWP-FA. The index for income was zero, indicating no effect. H5–H6 hypothesis was not supported. The index of moderated mediation for waist circumference with respect to age was not significant, as the confidence interval includes zero. This suggests that age did not significantly moderate the mediation effect of waist circumference. The index for income was zero, indicating no effect. H7–H8 hypothesis was not supported. The index of moderated mediation for BMI with respect to age was not significant, as the confidence interval includes zero. This suggests that age did not significantly moderate the mediation effect of BMI. The index for income was zero, indicating no effect. H9–H10 hypothesis was not supported.

## 4 Discussion

Food waste has recently emerged as a threat that has negative economic, social, and environmental consequences. The environmental and nutritional implications of food waste have attracted attention globally (29). There are many factors to consider in the relationship between food waste, nutrition and environmental sustainability (30). Consumers are estimated to be the most wasteful stage in the food supply chain (31). Therefore, this study aimed to determine the relationship between attitudes and behaviors toward preventing food waste and Green Eating behavior with the mediating role of the variables of BMI, waist circumference, and the frequency of actions to prevent food waste.

Social media usage and digital marketing interactions in Türkiye are the main factors that positively affect consumers' green food purchasing attitudes and behaviors (32). Many factors hinder the widespread adoption of the green diet concept. Among these are cultural eating habits, economic conditions, and technological limitations in existing food production systems. The strong influence of cultural preferences leads individuals to maintain traditional eating habits, while economic constraints, particularly for low-income groups, limit access to sustainable food products. Furthermore, technological limitations in food production and distribution increase the production costs of green foods, making it difficult for these products to be widely consumed (33, 34). Various innovative approaches are being explored to effectively meet sustainable nutritional needs on a global scale. These include biofortification, personalized nutrition strategies, and the development of alternative protein sources. Alternative proteins, particularly algae, plants, and green leaf proteins, can contribute to the widespread adoption of green diets by offering solutions that are both high in nutritional value and have a low environmental impact. These innovations are critical for overcoming the constraints of the current food system and promoting sustainable nutrition, both environmentally and healthily (35, 36). Therefore, the widespread adoption of green diets depends not only on changing individual preferences but also on the integrated implementation of economic accessibility, cultural adaptation, and technological innovation. Within this framework, the development of sustainable nutrition policies and the promotion of alternative proteins and innovative solutions such as biofortification will play a crucial role in ensuring global nutritional security in the future (37, 38).

The most important action to reduce food waste is to have a well-informed and organized consumer. Current evidence confirm that food waste is a complex issue that requires a broad approach to analysis considering several factors simultaneously (39). In this study, it is seen that age, educational status, income status, and body mass index levels are effective in preventing food waste. The effects of these demographic and anthropometric factors on food waste are widely discussed in the literature (31, 39). In the studies, regarding the age of the household members, young people waste more than older people. Concerning education, the studies show that a lower level of education matches a smaller quantity of food waste. This result is explained by the fact that people with a higher level of education are more likely to have a higher level of income and tend to spend and waste more (8, 40, 41). On the other hand, some individuals with low education levels may not be able to accurately estimate the percentage of wasted food (8). In addition, the higher the level of education reported behavior toward food waste prevention is known to be better (42). As for income, according to a study, at a lower income level, the level of waste is no lower than that found in high-income families (43). In another study found that households with higher income levels had higher levels of food waste (44). However, this study determined that high education and high-income levels were associated with Green Eating behavior. Similarly, in the literature, it is stated that education level and high socio-economic level are effective in sustainable eating habits (45, 46). Although overnutrition/obesity is seen as a component of food waste, eating more food than needed can be considered as a strategy to prevent food waste (47). In this study, the high food waste prevention behaviors of overweight and obese individuals may be associated with their behavior of consuming more than they need to prevent food waste.

This study's results showed that meat and eggs, cooked meals, and milk and dairy products were the most wasted food groups in women (Figure 1). In studies conducted in different societies, it is seen that the most wasted foods are cooked meals, especially vegetables, dairy products and meat, and meat products (48, 49). Storage conditions, incorrect cooking methods, preparation errors, and shopping mistakes are some of the reasons why meat and meat products, milk and dairy products, vegetables, and fruits are wasted. The wastage of cooked meals is caused by the amount of cooking, storage conditions after cooking, deficiencies in meal planning and menu management (50, 51).

Prevention of food waste and sustainable nutrition interact bidirectionally. Prevention of food waste is crucial for achieving sustainable nutrition and minimizing environmental impact. However, sustainable diets are key to reducing food waste and improving environmental sustainability (44, 52). In this study, behaviors to prevent food waste were found to be positively associated with Green Eating behavior. The results of another study reveal that the perceived value of sustainability has a highly significant effect on attitude, personal norms, and social norms on waste reduction. In addition, the adoption of environmentally friendly eating is positively influenced, in descending order, by personal norms, social norms, and attitudes toward waste reduction (53). Promoting sustainable eating habits is essential to the prevention of food waste and supporting a more resilient and equitable food system.

As obesity affects both individual dietary choices and broader food system dynamics, it can significantly impact sustainable nutrition efforts. Obesity often leads to increased consumption of processed and calorie-dense foods, which can strain environmental resources and undermine sustainability goals (54). The increased demand for high-calorie, low-nutrient foods associated with obesity can exacerbate environmental degradation by promoting intensive agricultural practices and higher greenhouse gas emissions (55). Additionally, the prevalence of obesity may inhibit the adoption of sustainable nutritional practices because individuals may prioritize immediate satiety and comfort over long-term environmental benefits. In this study, high waist circumference was associated with low Green Eating behavior. Addressing obesity by promoting healthier, plant-based diets not only benefits individual health, but also supports sustainable food systems by reducing the ecological footprint of food production and consumption. Therefore, tackling obesity is crucial for advancing sustainable nutrition and achieving environmental sustainability goals.

In addition, it is seen that waist circumference and BMI levels, which are obesity indicators, have no mediating effect on the relationship between attitudes and behaviors toward preventing food waste and Green Eating behavior. Overnutrition and obesity, the consumption of food above more than a balanced energy intake, should be considered a form of FW. At the same time, overnutrition contributes significantly to the overall environmental impact of the food chain (56). Although overnutrition/obesity is considered a component of food waste, eating more than needed can be considered a strategy to prevent food waste (47). This situation may be associated with the fact that while attitudes and behaviors toward preventing food waste can be observed in obese individuals, the level of Green Eating behavior as a sustainable nutrition model is low. However, it was determined that the indirect effect of the attitude and behavior for preventing food waste on Green Eating through BMI and waist circumference did not depend on income level and age. Although food wastage has been shown to be lower in obese people, it should be noted that obesity should not be seen as a strategy to prevent food wastage due to their low compliance with environmentally friendly diets and health concerns.

Our study confirms that attitudes and behaviors toward preventing food waste directly promote green eating, while obesity indicators such as BMI and waist circumference may weaken this effect. Engagement in food waste prevention mediates the translation of attitudes into sustainable eating behaviors, and these effects vary by age and income.

### 4.1 Practical implications

Targeted Campaigns: Develop age- and income-specific interventions to encourage food waste prevention and green eating behaviors.

Integration with Health Programs: Combine obesity prevention initiatives with sustainable diet promotion to reinforce green eating habits.

Community and Policy Support: Implement incentives and programs in schools, workplaces, and communities to normalize frequent food waste prevention and sustainable eating practices.

This concise framework links theoretical hypotheses to actionable strategies, guiding interventions that address both sustainability and health outcomes.

## 5 Conclusion

Attitudes and behaviors toward preventing food waste are directly related to green eating behavior, and obesity status may negatively affect this relationship. Attitudes and behaviors toward preventing food waste are also effective in the development of sustainable nutrition behaviors, and obesity prevention is important in strengthening this relationship.

## 6 Recomandations

*Education Programs Should Be Improved:*

- Design “food waste prevention and green eating” training programs for women with low education levels.
- Organize awareness-raising campaigns, especially for the 19–29 age group.

*Socioeconomic Factors Should Be Considered:*

- Conduct research on affordable, sustainable nutrition strategies suitable for low-income groups (e.g., consuming local/seasonal food).
- Conduct studies examining the impact of income support policies on green eating.

*Integrate Obesity Prevention Programs:*

- Because waist circumference negatively impacts green eating, integrate obesity prevention programs with sustainable eating.
- The lack of a meaningful indicator of BMI may necessitate the use of alternative health indicators (e.g., metabolic health).
- Age- and education-appropriate awareness campaigns: Prevent food waste and encourage sustainable eating behaviors through interactive training and information programs aimed at different age and education groups.
- Integrated approaches to obesity prevention: In obesity prevention programs, messages about healthy eating and reducing food waste should be delivered together, thus reinforcing healthy and sustainable eating habits.
- Community and policy-supported practices: Support practices that reduce food waste and promote green eating in schools, communities, and institutions; drive behavioral change through incentives, workshops, and rewards.

## Data Availability

The datasets presented in this study can be found in online repositories. The names of the repository/repositories and accession number(s) can be found in the article/supplementary material.
